# Determination of HOMO–LUMO Energy Levels of Carbon Dots via Electron Transfer Kinetics and Marcus Theory

**DOI:** 10.3390/molecules31081247

**Published:** 2026-04-09

**Authors:** Mengli Yang, Xiaoyu Yu, Yang Yang, Huiqi Shi, Bianyang He, Weishuang Li, Yaoyao Zhang, Lei Zhu

**Affiliations:** School of Chemistry and Materials Science, Hubei Engineering University, Xiaogan 432000, China; mlyang@hbeu.edu.cn (M.Y.); yxy070214@gmail.com (X.Y.); 081718y@gmail.com (Y.Y.); shq0124aa@outlook.com (H.S.); hby891019@hbeu.edu.cn (B.H.); liweishuang706@hbeu.edu.cn (W.L.)

**Keywords:** carbon dots, HOMO–LUMO energy levels, Marcus theory, electron transfer kinetics, fluorescence quenching

## Abstract

The precise determination of highest occupied molecular orbital (HOMO) and lowest unoccupied molecular orbital (LUMO) energy levels is critical for understanding the photophysical and photochemical properties of carbon dots (C-dots), which directly govern their performance in optoelectronic, catalytic, and sensing applications. However, the lack of distinct redox peaks in cyclic voltammetry (CV) curves of C-dots poses a major challenge to conventional energy level calculation methods. Herein, we propose a novel strategy to calculate the HOMO–LUMO energy levels of C-dots by combining electron transfer (ET) kinetics with Marcus theory. A series of quinones (electron acceptors, EAs) and ferrocene derivatives (electron donors, EDs) were employed to quench the fluorescence of C-dots, and the ET rate constants (K) were derived from fluorescence lifetime measurements. The CV curves of EAs and EDs provided their respective oxidation and reduction potentials, which were used as reference energy levels. The UV–Vis absorption spectra confirmed that the fluorescence quenching mechanism was dominated by ET rather than energy transfer. Based on Marcus theory, the free energy change (ΔG) of ET reactions was correlated with K, and the HOMO and LUMO energy levels of C-dots were calculated to be −1.84 V (vs. SCE) and +1.60 V (vs. SCE), respectively. This study not only provides a reliable method for determining the energy levels of C-dots without distinct redox peaks but also deepens the understanding of ET mechanisms between C-dots and small molecules. The proposed strategy is expected to be extended to other fluorescent nanomaterials with similar CV limitations.

## 1. Introduction

Carbon dots (C-dots), as a class of zero-dimensional carbon-based nanomaterials, have garnered enormous attention over the past two decades due to their unique properties, including tunable fluorescence emission, high photostability, low toxicity, and excellent solubility in various solvents [1,2,3,4,5]. These merits render C-dots promising candidates for applications in fluorescence sensing, bioimaging, photoelectrochemical devices, and photocatalysis [6,7,8]. The performance of C-dots in these fields is inherently determined by their electronic structure, particularly the HOMO and LUMO energy levels, which dictate the feasibility and efficiency of electron/energy transfer processes with other molecules or materials [9,10,11].

Conventional methods for determining the HOMO–LUMO energy levels of C-dots primarily rely on cyclic voltammetry (CV) and UV–Vis absorption spectroscopy [12,13]. The CV method calculates the HOMO level from the oxidation potential and the LUMO level from the reduction potential based on a reference electrode. However, many C-dots exhibit no distinct redox peaks in CV curves due to their amorphous structure, surface state complexity, or low electron transfer efficiency at the electrode interface, making this method inapplicable. The UV–Vis absorption method estimates the optical band gap (E) from the onset absorption wavelength but cannot directly provide the absolute energy levels of HOMO and LUMO [14,15]. Thus, developing an alternative method to accurately determine the HOMO–LUMO energy levels of C-dots with unclear CV redox peaks is highly desirable.

Fluorescence quenching based on ET has been widely used to study the interaction between C-dots and small molecules [16,17,18,19]. Sun et al. reported that photoluminescence of C-dots could be quenched by electron donors of N,N-diethylaniline (DEA, 0.88 V vs. NHE), indicating that C-dots behaved as excellent electron acceptors. Purkayastha et al. found that the addition of electron acceptor MV^2+^ can result in the fluorescence quenching of C-dots to various degrees, declaring that C-dots have achieved electron-donating capacity. When C-dots interact with EAs or EDs, ET can occur from excited-state C-dots to EAs (oxidative ET) or from EDs to excited-state C-dots (reductive ET), resulting in fluorescence quenching. The ET rate constant (K) can be obtained from fluorescence lifetime measurements, which is closely related to the free energy change (ΔG) of the ET reaction according to Marcus theory [20,21,22]. Marcus theory describes the relationship between K, ΔG, reorganization energy (λ), and temperature (T), providing a theoretical basis for correlating ET kinetics with energy levels [23].

In this work, we report a novel approach to determine the HOMO–LUMO energy levels of C-dots using ET kinetics and Marcus theory. Quinones (1,4-benzoquinone, tetrachloro-1,4-benzoquinone, etc.) and ferrocene derivatives (ferrocene, 1,1′-dimethylferrocene, etc.) were selected as EAs and EDs, respectively. The fluorescence quenching of C-dots by these molecules was confirmed to be ET-dominated via UV–Vis absorption spectroscopy and fluorescence lifetime measurements. The CV curves of EAs and EDs provided their reference energy levels. By fitting the K values with Marcus theory, ΔG for each ET reaction was obtained, and the HOMO and LUMO energy levels of C-dots were further calculated. This method overcomes the limitation of unclear CV redox peaks of C-dots and provides a new avenue for determining the electronic structure of fluorescent nanomaterials.

## 2. Discussion

### 2.1. Cyclic Voltammetry Measurement of C-Dots

**Figure 1:**
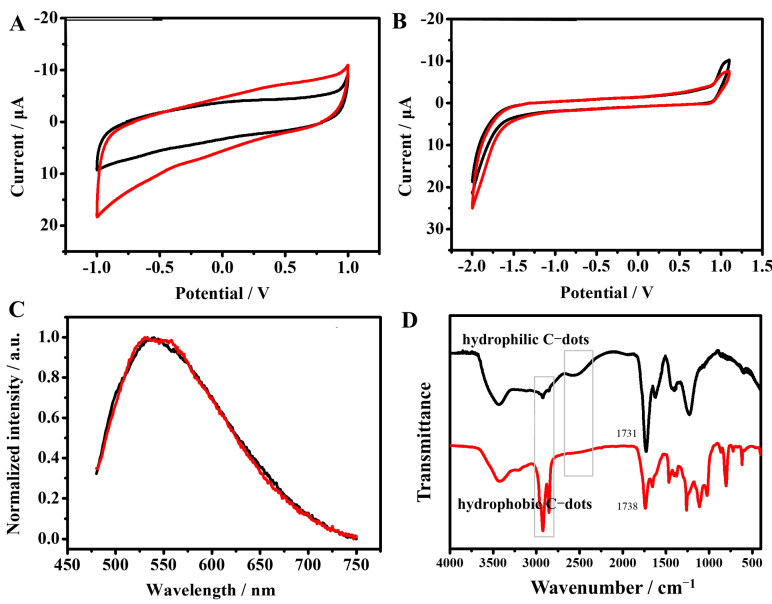
Cyclic voltammogram of a glassy carbon electrode in (**A**) 0.2 M pH 7.2 phosphate buffer and (**B**) CH_3_CN solution with Bu_4_NPF_6_ as a supporting electrolyte. Scan rate is 0.1 V s^−1^. (**C**) The PL spectra of hydrophilic C-dots and hydrophobic C-dots under the same excitation. The excitation wavelength is 460 nm. (**D**) FT-IR characterization of C-dots, the black line represent the hydrophilic C-dots and the red line represent the hydrophobic C-dots, the pesk about 2570 cm^−1^ represent the hydrogen-bonded stretching vibration peak of carboxyl groups, peaks at 2850–2960 cm^−1^ is attributed to the stretching and bending vibrations of methyl and methylene groups.

**Figure 2:**
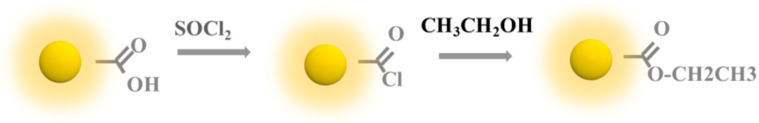
The scheme for esterification of C-dots.

TEM characterization shows that the C-dots are uniformly dispersed with an diameter of 2.1 ± 0.7 nm (Figure S1). The high-resolution C 1s spectrum was peak-fitted into four components, corresponding to graphitic carbons, alcoholic carbons, carbonyl carbons, and carboxyl carbons. The contents of these groups were calculated to be 58.4%, 17.1%, 17.8%, and 6.7%, respectively (Figure S2). The cyclic voltammetry of C-dots in 0.2 M deoxygenated phosphate buffer was displayed in Figure 1A. None of apparent oxidation or reduction peaks could be observed. The hydrophilic C-dots were transferred to hydrophobic C-dots by the esterification reaction with ethanol as outlined in Figure 2. The cyclic voltammetry of hydrophobic C-dots was conducted in CH_3_CN solution, with Bu_4_NPF_6_ as a supporting electrolyte (Figure 1B). Similarly, no redox peaks were observed. The reason may be likely attributed to the loss of the electrochemical activity center in C-dots. Fluorescence spectra of C-dots before and after esterification were also measured to evaluate the effect of surface modification on photoluminescence properties. The fluorescence emission peak position of C-dots remained almost unchanged at 538 nm, indicating that the esterification reaction did not alter the intrinsic fluorescence characteristics of C-dots, which is crucial for ensuring the reliability of subsequent fluorescence quenching experiments. The esterification reaction was characterized by Fourier transform infrared (FT-IR) spectroscopy. As shown in Figure 1D, the hydrogen-bonded stretching vibration peak of carboxyl groups at 2570 cm^−1^ (attributed to hydrophilic C-dots) disappeared after esterification. Meanwhile, the carbonyl (C=O) stretching vibration peak shifted to a higher wavenumber from 1731 cm^−1^ to 1738 cm^−1^, which is associated with the formation of ester carbonyl groups. Additionally, new characteristic peaks at 1261 cm^−1^ and 1112 cm^−1^ (corresponding to the C-O stretching vibration of esters) appeared in the FT-IR spectrum of hydrophobic C-dots. These spectral changes confirm the successful transformation of esterification reaction.

### 2.2. Fluorescence Quenching Mechanism

**Figure 3:**
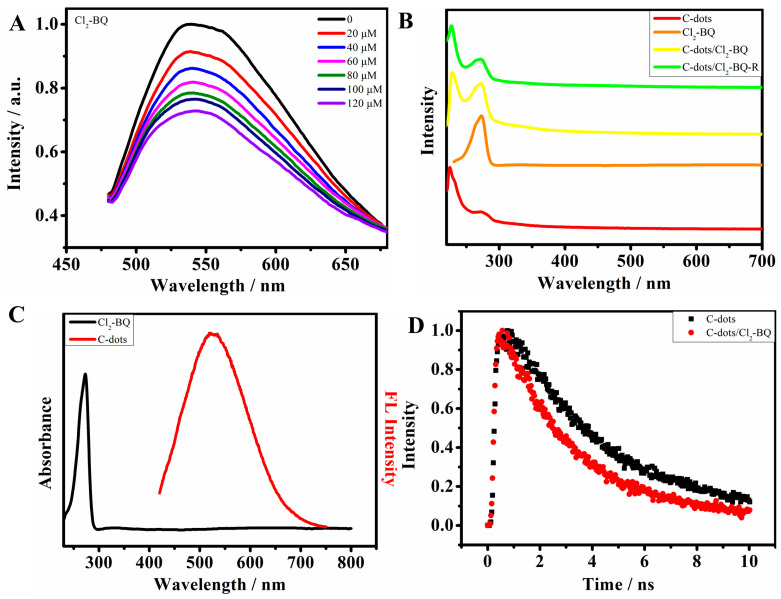
(**A**) The PL quenching spectra of C-dots by Cl_2_-BQ. (**B**) UV–Vis absorption spectra of C-dots, Cl_2_-BQ, C-dots/Cl_2_-BQ, and the light irradiation of the mixture. (**C**) UV–Vis absorption peak of DCBQ and the PL emission spectrum of C-dots. (**D**) The fluorescence decay curves of C-dots before and after adding Cl_2_-BQ.

The C-dots used in our following experiments are all hydrophobic C-dots in CH_2_Cl_2_. When DCBQ (Cl_2_-BQ, one of the EAs) was added to C-dots (Figure 3A), the fluorescence intensity gradually decreased with increasing Cl_2_-BQ concentration, indicating effective fluorescence quenching. To clarify the quenching mechanism, UV–Vis absorption spectra of C-dots, Cl_2_-BQ, and their mixtures were measured. As shown in Figure 3B, the absorption spectrum of the mixture is simply the superposition of the individual spectra of C-dots and Cl_2_-BQ, with no new absorption peaks appearing [24]. Furthermore, the light irradiation of the mixture of C-dots and quinones does not result in changes of the absorption spectrum, which exclude the process of photochemical degradation in C-dots. Additionally, the UV–Vis absorption peak of Cl_2_-BQ (λ = 270 nm) does not overlap with the fluorescence emission peaks of C-dots (Figure 3C), ruling out the possibility of Förster resonance energy transfer (FRET), which requires spectral overlap between the donor emission and acceptor absorption [25]. Fluorescence lifetime measurements were further performed. The fluorescence decay curves of C-dots before and after adding Cl_2_-BQ were fitted with a double-exponential function (Figure 3D). The average fluorescence lifetime (*τ*_avg_) of C-dots is 4.76 ns (Table 1), and it decreases to 4.03 ns after adding 20 mM Cl_2_-BQ, indicating the presence of dynamic quenching. Notably, a marked difference was observed between the intensity-based Stern–Volmer constant and the lifetime-based Stern–Volmer constant. The *K*_sv_ calculated from the lifetime (*τ*_0_/*τ* = 1 + *K*_sv_[Q]) is 10.9 M^−1^, while the *K*_sv_ calculated from the intensity (*I*_0_/*I* = 1 + *K*_sv_[Q]) is 3295 M^−1^. The value of K_Sv_(*I*) is significantly larger than K_sv_(*τ*), which is a typical indicator of mixed quenching involving both static quenching and dynamic quenching. The quenching rate constant derived from fluorescence lifetime is *k*_q_ = *K*_sv_/*τ*_0_ = 2.3 × 10^9^ M^−1^·s^−1^, much larger than 10^7^ M^−1^·s^−1^ (the threshold for dynamic quenching by diffusion) [26]. This confirms that dynamic electron transfer is the intrinsic quenhcing pathway responsible for the Marcus kinetic analysis. Since static quenching only affects fluorescence intensity but not the excited-state lifetime, only the lifetime-derived k_q_ was used for subsequent electron transfer kinetics and Marcus theory fitting, ensuring that the analysis reflects the intrinsic dynamic electron transfer process.

### 2.3. Redox Potentials of Electron Acceptors and Donors

**Figure 4:**
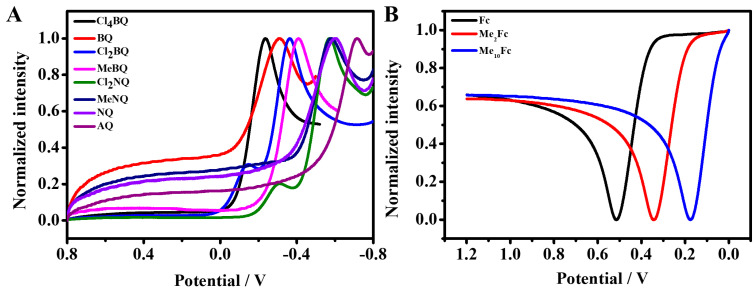
(**A**) CV measurements of the quinones in CH_2_Cl_2_ solution. (**B**) CV measurements of the ferrocene derivatives in CH_2_Cl_2_ solution (E_1/2_ in V vs. Ag wire).

CV measurements were carried out to determine the redox potentials of EAs and EDs (Figure 4), which serve as reference energy levels for calculating the HOMO–LUMO levels of C-dots. For EAs (quinones), the reduction potential (E) corresponds to the one-electron reduction of quinones to semiquinone radicals; for EDs (ferrocene derivatives), the oxidation potential (E) corresponds to the one-electron oxidation of ferrocene to ferrocenium ions. The three-electrode system was applied to the measurements, and the referrence electrode we used was Ag wire. All potentials were converted to potentials relative to SCE by reducing 0.03V, where 0.03 V is the open-circuit voltage of Ag relative to SCE. The calibrated reduction potentials of EAs are listed in Table 2 and the oxidation potentials of EDs are listed in Table 3. For example, the reduction potential of Cl_2_-BQ is −0.39 V vs. SCE, and the oxidation potential of Fc is 0.48 V vs. SCE.

### 2.4. Electron Transfer Kinetics and Marcus Theory Fitting

**Scheme 1:**
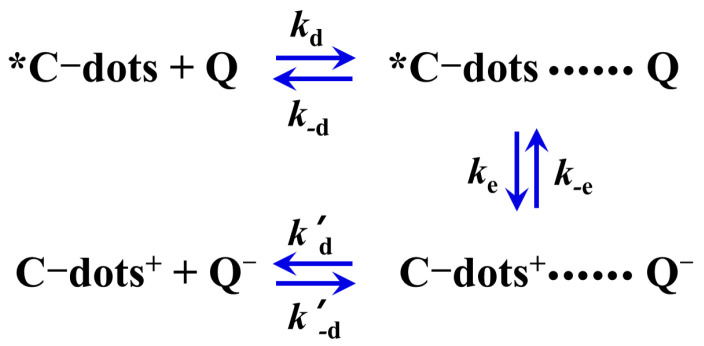
Kinetics scheme for electron transfer quenching.

An electron transfer quenching process can be discussed on the basis of the kinetics scheme shown in Scheme 1. The quenching rates of *k_q_* can be written as

(1) $$k_{q}=\frac{k_{d}}{1+\frac{k_{\text{-}d}}{k_{e}}+\frac{k_{\text{-}d}}{k_{\text{-}d}^{\prime }}\frac{k_{\text{-}e}}{k_{e}}}$$

where *k_d_* is diffusion rate constant, *k*_-d_ and *k*′_-d_ are the rate constant for dissociation of the complex, *k_e_* and *k*_-e_ are the unimolecular for forward and backward electron transfer processes, the ration of *k_e_* and *k*_-e_ are determined by Δ*G* (Equation (2)), where Δ*G* is the free energy change of the electron transfer step and is related to the reduction potentials of C-dots and quinones *Q* (Equation (3)). *F* is Faraday constant, *E*_00_(*C-dots/C-dots) is the one-electron potential corresponding to the PL band gap of C-dots, and *w* is the electrostatic work accounts for Coulomb interactions between the pairs after electron transfer.

(2) $$\frac{k_{\text{-}e}}{k_{e}}=e^{(∆G/\mathrm{RT})}$$

(3) $$∆G=F\left[E\left(C\text{-}\mathrm{dot}s^{+}/C\text{-}\mathrm{dots}\right)-E_{00}\left(*C\text{-}\mathrm{dots}/C\text{-}\mathrm{dots}\right)-E\left(Q/Q^{-}\right)\right]+w$$

In traditional Marcus theory, k_e_ can be expressed by Equation (4), where k is the electronic transmission coefficient, ν_n_ is the nuclear factor, and ΔG* is the free activation energy. In Equation (5), ΔG*(0) is the intrinsic barrier, a parameter related to the amount of distortion of both the inner coordinates and the outer solvation shells accompanying electron transfer. The expression of activation free energy in Equation (5) is derived from the classical Marcus electron transfer theory under weak electronic coupling conditions, which is suitable for describing the activation process of homogeneous electron transfer reactions. Here, ΔG* represents the total activation free energy, ΔG*(0) is the intrinsic activation barrier corresponding to the reorganization energy λ, and ΔG is the Gibbs free energy change of the electron transfer reaction. This formula is used to describe the nonlinear relationship between activation free energy and driving force in electron transfer reactions. A constant reorganization energy λ (represented as ΔG*(0)) was used for all quenchers in the fitting process. This treatment is standard in Marcus analysis for a fixed donor (C-dots) and a series of structurally similar acceptors/donors. Analysis shows that small variations in λ (±0.05 eV) lead to an error of <0.1 eV in the calculated energy levels, which is acceptable for ensemble characterization.

According to the standard Marcus theory, the activation free energy of direct electron transfer can be expressed as: ΔG* = (λ + ΔG + w)^2^/4λ where λ is the total reorganization energy, ΔG is the reaction free energy, and w is the electrostatic work term generated by charge separation after electron transfer. For backward electron transfer, the electrostatic work term w is negligible, and the activation free energy is simplified to ΔG* = (λ + ΔG)^2^/4λ. This asymmetry between direct and backward electron transfer arises from the Coulomb interaction between the charged products in the forward reaction, which has been considered in the kinetic analysis.

It should be emphasized that the present study is based on the non-adiabatic electron transfer regime under weak electronic coupling, which is suitable for solution-phase electron transfer between C-dots and small molecular quenchers. For adiabatic electron transfer reactions under strong electronic coupling, especially for electron transfer processes on semiconductor electrode surfaces, a different theoretical formalism is required, as systematically established by Schmickler et al. [27].

(4) $$k_{e}={\mathrm{kv}}_{n}\exp(-∆G^{*}/\mathrm{RT})$$

(5) $$∆G^{*}=∆G+\frac{∆G^{*}\left(0\right)}{\ln2}\ln\left(1+\exp\left(\frac{-∆G\;\ln2}{∆G^{*}\left(0\right)}\right)\right)$$

If we consider a series of homogeneous electron transfer reactions such as those between a group of oxidants having the same size, shape, electronic structure, and electric charge, and a group of reductants also having the same size, shape, electronic structure, and electric charge, we may assume throughout the series that the reaction parameters *k*_d_, *k*_-d_, *k*′_-d_ in Equation (2), *k* in Equation (4), and ΔG*(0) in Equation (5) are constant. This approximation is widely used in Marcus kinetic analyses of a series of structurally similar quenchers with the same donor/acceptor. Although the quinone and ferrocene derivatives differ slightly in polarizability and steric hindrance, all quenchers are small neutral molecules with similar size and charge, leading to negligible differences in reorganization energy and diffusion behavior. This approximation does not affect the reliable determination of HOMO–LUMO levels, which is the main purpose of this work. Under these assumptions, *k*_q_ in Equation (1) is only a function of the free energy change, i.e., of the redox potential of the reaction partners. Based on the above equation, a plot of log k vs. ΔG consists of (i) a plateau region for sufficiently exergonic reactions, (ii) an Arrhenius type linear region (slope 1/(2.3RT)) for sufficiently endergonic reactions, and (iii) an intermediate region (centered at Δ*G* = 0) in which k increases in a complex but monotonous way as Δ*G* decreases.

**Figure 5:**
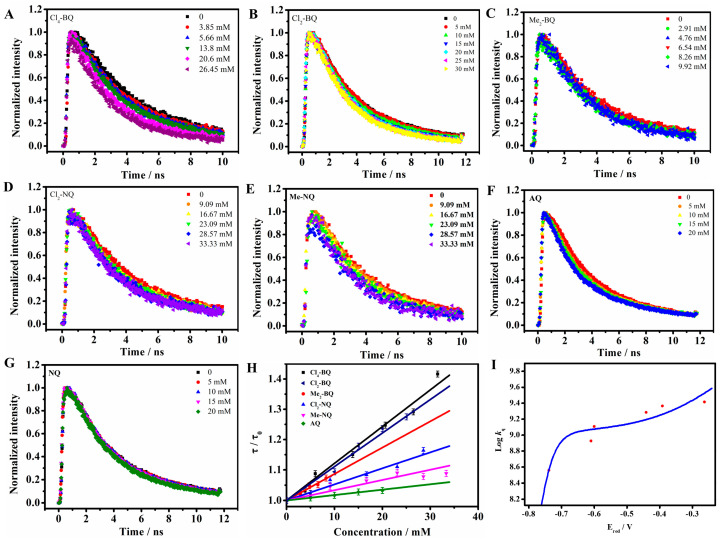
(**A**–**G**) The fluorescence decay curves of C-dots with different concecntration of quinones. (**H**) The relationship of τ_0_/τ with the concentration of quenchers. (**I**) Marcus theory fitting curves of Logk_q_ vs. Ered (in V vs. SCE) for oxidative ET (C-dots → EAs). The blue solid lines are the fitting results.

Rates of electron transfer from the various quinones to C-dots were measured by time-revolved fluorescence spectra (Figure 5A–G). Table 2 lists the measured rate constants for quinones. Figure 5I shows the respective plots of logarithms of these rate constants vs. the potential of quinones. From the best fitting of the data reported in Figure 5I, two parameters are obtained: the value of ΔG*(0) is 0.194 eV; *kν*_n_ is 8.99 × 10^7^. *E*_00_(*C-dots/C-dots) is estimated from the maximum of the emission band of 2.34 eV, so the value of E(C-dots^+^/C-dots) is +1.60 V vs. SCE.

**Figure 6:**
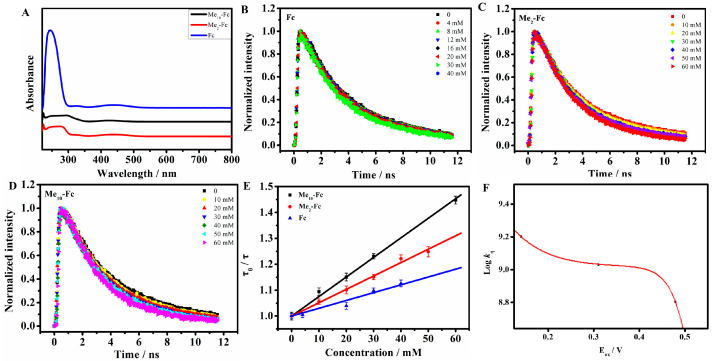
(**A**) Normalized absorption spectra of Fc (blue line), Me_2_-Fc (black line), and Me_10_-Fc (red line). (**B**–**D**) The fluorescence decay curves of C-dots with different concecntrations of ferrocene derivatives. (**E**) The relationship of τ_0_/τ with the concentration of ferrocene derivatives. (**F**) Marcus theory fitting curves of logkq vs. Eox (in V vs. SCE) for reductive ET (EDs → C-dots). The red solid lines are the fitting results.

Electron transfer in the reverse direction can be observed whenever the quenchers have sufficient energy to be denoted. Electron donor molecules, i.e., ferrocene derivatives ferrocene (Fc), 1,1′-dimethyl-ferrocence (Me_2_-Fc), and decamethylferrocene (Me_10_-Fc), were chosen in this experiment. As shown in Figure 6A, the absorption spectra of ferrocene and its derivatives have weak absorption in the range of 400–500 nm, so we choose fluorescence lifetime *τ*_0_/*τ* to calculate the *k*_q_ value, as lifetime would not be influenced by the absorption of Fc and C-dots. FRET can also be excluded, as Fc has a triplet excited state that lies at 1.78 eV [28], while the excition of C-dots occurs at higher energy levels. Same as before, as Fc was added into C-dots, the PL lifetime of C-dots decreased with the increase in quencher concentration (Figure 6B–D). The combination of decreased PL lifetimes and absorption spectra, cited below, is sufficient to ensure that only the electron transfer step occurs to a measureable extent. By ploting τ_0_/τ vs. the quencher concentration (Figure 6E), the *k*_q_ values were calculated in Table 3. In the reductive electron transfer, the free energy change can be estimated by Equation (6). Through the best fitting of curve in Figure 6F, the potential of $E\left(C\text{-}\mathrm{dots}/C\text{-}\mathrm{dot}s^{-}\right)$ is −1.84 V.

(6) $$∆G=F\left[E({\mathrm{Fc}}^{+}/\mathrm{Fc})-E\left(C\text{-}\mathrm{dots}/C\text{-}\mathrm{dot}s^{-}\right)-E_{00}\left(*C\text{-}\mathrm{dots}/C\text{-}\mathrm{dots}\right)\right]+w$$

**Figure 7:**
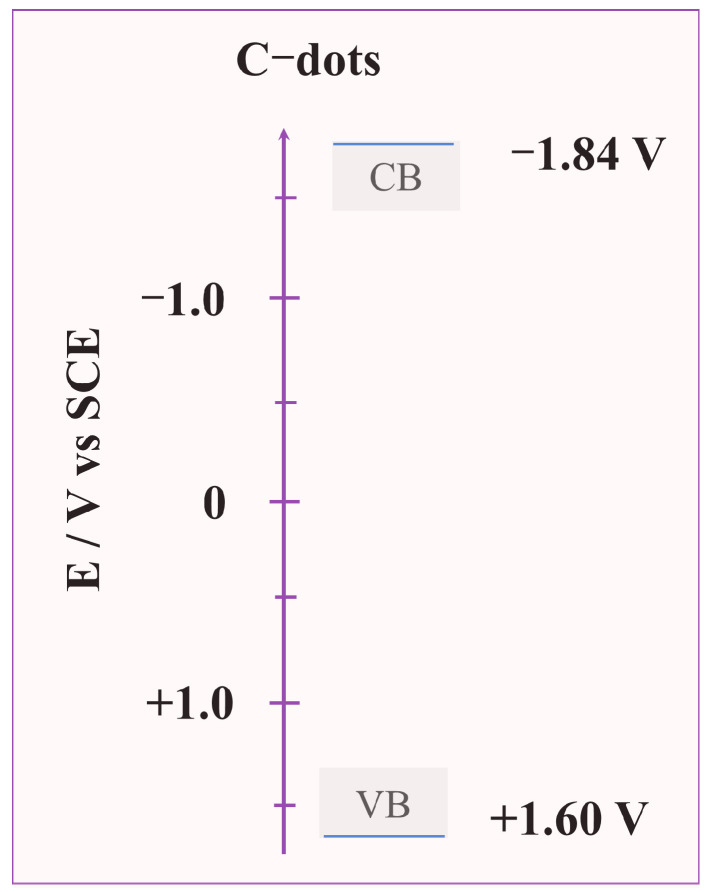
Energy level diagram of C-dots versus a saturated calomel electrode (SCE). The CB (LUMO) and VB (HOMO) levels were determined to be −1.84 V and +1.60 V vs. SCE, respectively.

Based on the fitted ΔG values and the redox potentials of EAs/EDs, the HOMO and LUMO energy levels of C-dots were calculated. The average LUMO level of C-dots calculated from different EAs is −1.84 V vs. SCE, and the average HOMO level calculated from different EDs is +1.60 V vs. SCE (Figure 7). The optical band gap (E) is calculated as 3.44 eV, which is consistent with the band gap derived from the UV–Vis absorption onset (3.54 eV) (Figure S1), confirming the accuracy of the calculation.

The reliability of our method is supported by comparison with recent DFT benchmark studies [29], which evaluated 15 functionals for predicting HOMO–LUMO gaps in organic materials and identified several high-accuracy methods for carbon-based π-conjugated systems. Our experimentally determined energy levels and optical gap (3.44 eV) are consistent with the ranges predicted by these benchmark functionals for oxygenated carbon nanostructures. Notably, our method provides ensemble-averaged experimental energy levels that reflect the true heterogeneity and solution environment of C-dots, whereas DFT calculations typically rely on idealized single models. The two approaches are complementary, and the consistency between them confirms the reliability of our proposed strategy.

## 3. Experimental Section

### 3.1. Synthesis of C-Dots

Synthesis of C-dots: C-dots was performed by chemical oxidation of carbon fibers. CFs (0.4 g) were added to a boiled mixed acid solution (30 mL, V_H2SO4_:V_HNO3_ = 1:3) and refluxed for 2 h [30]. The corresponding solution was neutralized with NaHCO_3_ until the pH reached 3~4. The resulting solution was filtered by BIOSHARP membrane filters (0.22 µm) and then further dialyzed for 7 days to remove small-molecule impurities. Afterwards, the C-dots solution was ultrafiltered successively through Millipore centrifugal filter devices with five different molecular-weight cut-off (MWCO) membranes (i.e., 100, 50, 30, 10, and 3 kDa). Finally, the <3 KDa was collected and freeze-dried to obtain hydrophilic C-dots powder for the analysis.

Transfermation of hydrophilic C-dots to hydrophobic C-dots: In this study, the quenching experiments were conducted in the CH_2_Cl_2_ solution. Therefore, C-dots need to be transferred from hydrophilic to lipophilic. The functionization of C-dots was the same procedure as those reported previously [31]. C-dots were treated with thionyl chloride followed by esterification with ethanol. Finally, the modified C-dots were rotary evaporated and dispersed in CH_2_Cl_2_ for further use. The PL properties of the modified C-dots in CH_2_Cl_2_ were investigated. Under the same excitation, the PL fluorescence peak position remained the same as C-dots in aqueous phase, which implied that the modification do not influence the predominant emissive sites.

### 3.2. Fluorescence Quenching Experiments

Stock solutions of EAs (BQ, Cl_4_-BQ, Cl_2_-BQ, NQ, Cl_2_-NQ, AQ) and EDs (Fc, Me_2_-Fc, Me_10_-Fc) were prepared at 0.1 M in CH_2_Cl_2_, with the final working concentrations of each quencher explicitly reported in the corresponding figures. Hydrophilic C-dots were dispersed in ultrapure water to a final concentration of 0.2 mg mL^−1^; hydrophobic C-dots were dispersed in CH_2_Cl_2_ to 0.2 mg mL^−1^.

For quenching experiments, C-dots dispersion was added to different volumes of EA/ED stock solutions. After each addition, the mixture was stirred for 5 min, and then the fluorescence spectrum and lifetime were measured. The Stern–Volmer quenching constant (*K*) was calculated from the Stern–Volmer equation *I*_0_/*I* = 1 + *K*_sv_[Q], where I_0_ and I are the fluorescence intensities in the absence and presence of quencher (Q), respectively, and [Q] is the quencher concentration. The ET rate constant *k*_q_ was derived as *k*_q_ = *K*_sv_/*τ*_0_, where τ_0_ is the average fluorescence lifetime of C-dots in the absence of quencher.

## 4. Conclusions

In summary, we have developed a novel method to determine the HOMO–LUMO energy levels of C-dots with unclear CV redox peaks by combining ET kinetics and Marcus theory. The fluorescence quenching of C-dots by quinones (EAs) and ferrocene derivatives (EDs) was confirmed to be ET-dominated via UV–Vis absorption spectroscopy and fluorescence lifetime measurements. The redox potentials of acceptors and donors were obtained from CV measurements, serving as reference energy levels. By fitting the ET rate constants with Marcus theory, the free energy changes of ET reactions were determined, and the HOMO and LUMO energy levels of the C-dots, referenced to a SCE, were determined to be +1.60 V and −1.84 V, respectively. The calculated optical band gaps are consistent with those derived from UV–Vis absorption spectra, verifying the accuracy of the method. This study overcomes the limitation of conventional CV methods for C-dots with ambiguous redox peaks and provides a reliable alternative for determining the electronic structure of fluorescent nanomaterials. The proposed strategy not only deepens the understanding of ET mechanisms between C-dots and small molecules but also expands the application scope of Marcus theory in nanomaterial research. Future work will focus on optimizing the method for C-dots with different compositions and structures, as well as exploring the correlation between energy levels and the performance of C-dots in sensing and photocatalysis.

## Figures and Tables

**Table 1:** Multiexponential fitting for femtosecond time-resolved absorption dynamics.

	*τ*_1_ (ns)	*τ*_2_ (ns)	*τ*_avg_ (ns)
C-dots	5.82 (76.0%)	1.37 (24.0%)	4.76

**Table 2:** Reduction potentials of the electron acceptors in CH_2_Cl_2_ solution and rate constants of the quenching of C-dots upon addtion of quinones.

Quenchers	*K*_sv_	E_red_ (V vs. SCE)	Log *k*_q_
Cl_4_-BQ	12.15	−0.26	9.41
Cl_2_-BQ	11	−0.39	9.36
Me_2_-BQ	9	−0.44	9.28
Cl_2_-NQ	6	−0.60	9.10
Me-NQ	4	−0.61	8.92
NQ	—	−0.63	—
AQ	1.7	−0.74	8.55

**Table 3:** Oxidation potentials of the electron acceptors in CH_2_Cl_2_ solution and rate constants of the quenching of C-dots upon addtion of ferrocene derivatives.

Quenchers	K_sv_	E_ox_/V	Log *k*_q_
Fc	3.02	0.48	8.80
Me_2_-Fc	5.16	0.31	9.03
Me_10_-Fc	7.75	0.14	9.20

## Data Availability

The original contributions presented in this study are included in the article/Supplementary Material. Further inquiries can be directed to the corresponding authors.

## Supplementary Materials

The following supporting information can be downloaded at: https://www.mdpi.com/article/10.3390/molecules31081247/s1, Figure S1: (a) The TEM of C-dots and (b) the statistical size analysisof the C-dots by TEM; Table S1: The relative content of different oxygen-containing functional groups of C-dots; Figure S2: The high resolution C 1s spectra of C-dots; Figure S3: Tauc plots of C-dots. The optical band gap is derived from the intercept of the linear portion of the Tauc plot with the x-axis.
