# Supplementary material for: Determination of HOMO–LUMO Energy Levels of Carbon Dots via Electron Transfer Kinetics and Marcus Theory
Source: Molecules. 2026 Apr 9;31(8):1247. doi: 10.3390/molecules31081247 (PMC13118674; doi:10.3390/molecules31081247)
Supplement: Supplementary file 1 [file molecules-31-01247-s001.zip › molecules-4201675-supplementary.pdf]

Supporting Information

Materials

Carbon fibers were purchased from Shanghai Tansu Manufactory. HNO_3 (65%~68%), while H_2SO_4 and $NaHCO_3$ were purchased from local suppliers. 1,4-benzoquinone (BQ), tetrachloro-1,4-benzoquinone (Cl_4-BQ), 2,5-dichloro-benzoquinone (Cl_2-BQ), 1,4-naphthoquinone (NQ), 2,3-dichloro-1,4-naphthoquinone (Cl_2-NQ), 9,10-anthraquinone (AQ), ferrocene (Fc), 1,1′-dimethylferrocene (Me_2-MFc), and decamethylferrocene (Me_{10}-Fc) were purchased by Sigma Aldrich, phosphate-buffered saline (PBS, 0.01 M, pH 7.4) and acetonitrile (CH_3CN, HPLC grade) were purchased from Aladdin Reagent Co., Ltd. (Shanghai, China). All chemicals were used without further purification. Deionized water (18.2 MΩ·cm) was used throughout the experiments.

Characterization

PL spectra were recorded by Horiba Jobin Yvon Fluorolog-3 fluorescence spectrometer; the fluorescence emission spectra of CNDs before and after esterification were measured with an excitation wavelength of 460 nm. The UV–Vis absorption spectrum was recorded on Shimadzu UV-2550 UV–Vis spectrometer in the range of 200–800 nm. Fourier transform infrared (FT-IR) spectra were performed on Thermo Nicolet 360 FT-IR Spectrophotometer in the range of 4000–400 cm using the KBr pellet method. Fluorescent lifetime was recorded on another fluorescence spectrometer with a time-correlated single-photon counting (TCSPC) module (Edinburgh Photonics FLS920), with 400 nm pulse laser excitation. CV measurements were carried out on a CHI 660E electrochemical workstation (Shanghai Chenhua Instrument Co., Ltd., China) with a three-electrode system: glassy carbon working electrode (GCE, diameter 3 mm), platinum counter electrode, and Ag wire reference electrode. For hydrophilic systems, PBS (0.01 M, pH 7.4) was used as the electrolyte; for hydrophobic systems, CH_3CN containing 0.1 M tetrabutylammonium hexafluorophosphate (TBAPF) was used as the electrolyte.

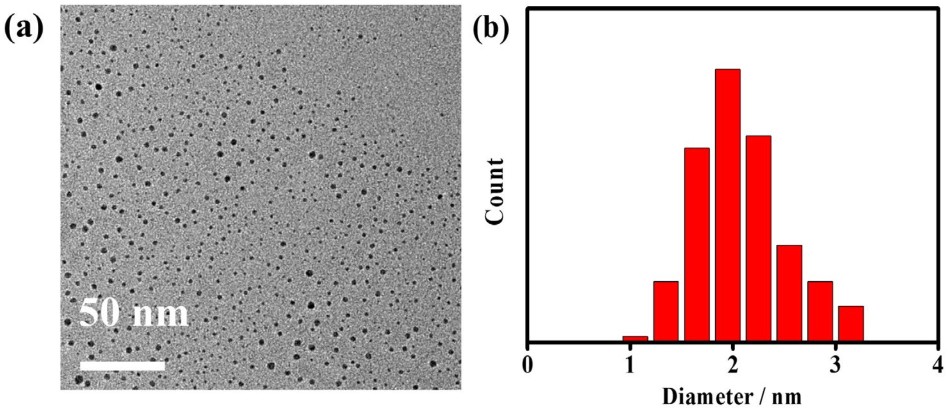

Figure S1 (a) The TEM of C-dots and (b) the statistical size analysis of the C-dots by TEM.

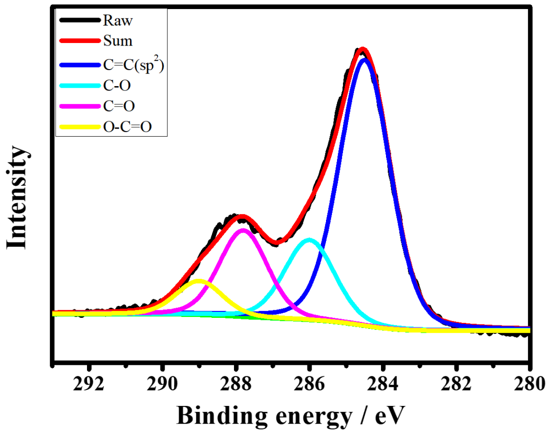

Figure S2 The high-resolution C 1s spectra of C-dots.

Table S1 The relative content of different oxygen-containing functional groups of C-dots.

	C=C	*C-O*	*C=O*	*-COO*
C-dots	58.4%	17.1%	17.8%	6.7%

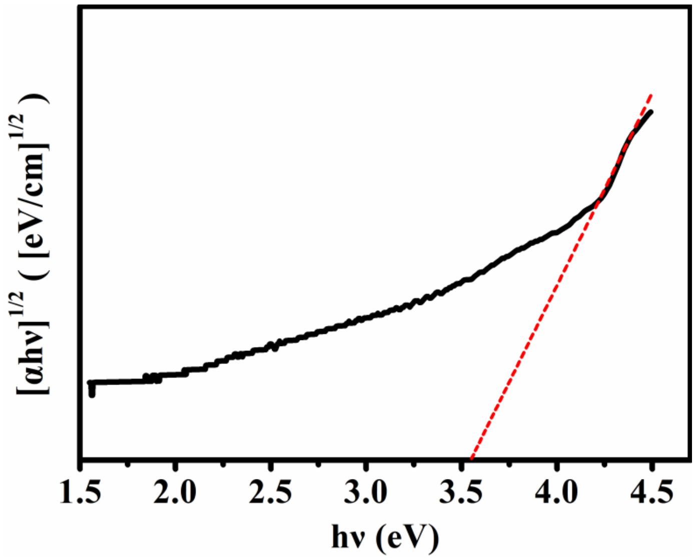

Figure S3 Tauc plots of C-dots. The optical band gap is derived from the intercept of the linear portion of the Tauc plot with the x-axis.

$$k_d = \frac{8RT}{3\eta}\frac{W_r/RT}{e^{W_r/RT}-1} \qquad \text{(S1)}$$

$$W_r = \frac{Z_D Z_A N e^2}{\varepsilon r\left(1+\sqrt{Ar\ \mu}\right)} \qquad \text{(S2)}$$

$$k_{-d} = k'_{-d} = \frac{2kT}{\pi r^3 \eta}\left(\frac{W_r/RT}{1-e^{-W_r/RT}}\right) \qquad \text{(S3)}$$
